# Internet Use for Creative Purposes and Its Correlation with Perceived Usefulness, Computer Anxiety, and Emotional Intelligence: The Intermediary Effect of the Perceived Ease of Use

**DOI:** 10.3390/bs15020221

**Published:** 2025-02-16

**Authors:** Kurtuluş Demirkol, Sena Esin İmamoğlu, Şaziye Serda Kayman, Salih Zeki İmamoğlu, Serhat Erat

**Affiliations:** Faculty of Business Administration, Gebze Technical University, 41400 Gebze, Türkiye; imamogluesin@gmail.com (S.E.İ.); skayman@gtu.edu.tr (Ş.S.K.); imamoglu@gtu.edu.tr (S.Z.İ.); erat@gtu.edu.tr (S.E.)

**Keywords:** computer anxiety, emotional intelligence, creativity, perceived ease of use, perceived usefulness

## Abstract

Although researchers have shown great interest in the antecedents and consequences of internet use due to the internet becoming a part of daily life, there is a gap in the literature regarding the factors that affect teachers’ use of the internet for creative purposes. This study aims to empirically examine teachers’ use of the internet for creative purposes and explores its relationship with emotional intelligence, computer anxiety, and the perceived ease of use of the internet. Furthermore, the possible intermediary effect of the perceived ease of use of the internet on creativity among these variables is also empirically investigated. In this context, data were obtained from 264 teachers in the Marmara Region in Turkey using a survey method. To test hypothesized relationships, structural equation modeling was conducted. Findings revealed that computer anxiety has a negative effect on creativity, while perceived usefulness, emotional intelligence, and the perceived ease of use have a positive effect. Our results also supported the partial mediating role of the perceived ease of use in the relationships between emotional intelligence and creativity, as well as between perceived usefulness and creativity, and the full mediating role of the perceived ease of use in the relationship between computer anxiety and creativity. Therefore, this research extends teachers’ understanding of technology acceptance and creativity by linking the two. Moreover, research findings provide important information to shape educational policies or professional development programs on the basis of digital education and offer a different approach to educators.

## 1. Introduction

The invention and development of computers and the internet during the second half of the 20th century radically changed not only daily life but also business life (Powell, 2013; Lin & Ma, 2022; Mohammed & Ralescu, 2023). The benefit of the internet as a powerful communication and information medium cannot be ignored (Roy, 2009), since it provides individuals with different means to fulfill their business or daily life requirements (Apandi et al., 2023). Moreover, the internet offers individuals the opportunity to access unlimited sources of information, thus becoming a source of creativity and changes creativity concepts in every respect (Lee, 2015). Workplace creativity is characterized by the creation and implementation of original and valuable ideas, aimed at solving problems and seizing new opportunities in the environment (Madrid & Patterson, 2016). Adding technology to the equation, Chen (2024) defines creativity as “generating new or previously unknown ideas or using advanced technologies to create dynamic, interactive and immersive learning environments that provide an in-depth understanding of known ideas in a new way”. On the other hand, Walia (2019) made a dynamic definition of creativity being “an act sparking out of a perception of the environment that acknowledges a certain disequilibrium, resulting in productive activity that challenges patterned thought processes and norms, and gives rise to something new in the form of a physical object or even a mental or an emotional construct”. Since creativity is a concept that can be evaluated in terms of both process and output (Giancola et al., 2022), Green et al. (2024) stated that an individual can be evaluated as creative not only by their outputs but also by whether they produce their current outputs through a creative process. Oldham and Da Silva (2015) suggested that for employees to produce creative ideas, access to new and diverse information, such as perspectives and ideas, full commitment to the job and the role in the workplace, and a feeling of support must be provided, and technology has the potential to facilitate these conditions. In addition to providing access to information, internet use also affects memory, future information access preferences, and metacognition (Oliva & Storm, 2023). This situation points to the importance of internet use for creative purposes and makes it necessary to understand how this can be possible. Although there are studies examining the relationship between the internet, social media, and various information technologies with creativity, there is a gap in the literature in terms of revealing the factors that affect internet use for creative purposes. 

Today’s world of education requires teachers to reach their students in more effective and diverse ways. Especially in the post-pandemic era, the internet, which facilitates access to educational resources, has become the new educational norm, enabling various forms of learning. Internet adoption has also been instrumental in providing flexible learning opportunities, allowing students to interact with content at their own pace and convenience (Ni et al., 2023). Teachers are expected to effectively select useful information from the pool of available knowledge and apply it effectively in both their professional and personal lives. Therefore, teachers need excellent technical preparation and adequate skills to adapt to changing educational requirements (Y. Li et al., 2023). The internet is not only a platform for the delivery of educational content, but also a facilitator of innovative educational practices that can improve teaching and learning outcomes in various contexts. The rapid integration of information and communication technologies (ICTs) into education and daily life and the development of the global information society have made it necessary for individuals to develop digital competencies necessary for participation in society. These skills are also necessary for educational institutions to keep pace with developments and follow innovative methods and processes (Yu et al., 2024). Thus, it is crucial to determine the factors that affect teachers’ willingness to adopt the use of the internet and guide them to adopt more innovative and student-centered approaches to use technology for productive purposes (Becker & Ravitz, 1999; Afari et al., 2023).

Emotional intelligence is crucial for future education professionals because it enables them to regulate both their own emotions and those of others, helping them navigate the challenges that they may encounter in their careers. Promoting emotional intelligence in educational settings can lead to increased personal well-being and academic performance (Valverde-Janer et al., 2023). Emotional intelligence is the capacity to recognize and assess one’s own emotions as well as those of others, to manage emotions effectively, and to use this emotional awareness to guide thinking and behavior in order to achieve positive outcomes (Dong et al., 2022). Sternberg (1985) suggested that emotional intelligence enables people to navigate the everyday environment more effectively and act creatively. Research in the literature also indicates a link between emotional intelligence and creativity, suggesting that individuals with high emotional intelligence tend to be more positive and creative (Dong et al., 2022). Moreover, previous studies have paid considerable attention to the relationship between internet use and emotional intelligence, but they have generally focused on the relationship between emotional intelligence and internet addiction or problematic internet use (Fernández-Martínez et al., 2023; Hoang et al., 2023; Sechi et al., 2021; Vu et al., 2022). In other words, although past studies have addressed the relationship between emotional intelligence and the negative aspects of internet use, there is not enough information about its relationship with internet use for creative purposes.

The key decisive factor affecting individuals’ attitudes about any technology, and the motivator to use it, is agreed that it is the perceived ease of use (Huang & Chueh, 2021) which refers to individuals’ perception of using a particular technology easily (Akdim et al., 2022). When the perceived ease of use is high, individuals think that they can use a certain technology easily and will not have problems using it (Price-Howard & Lewis, 2023). Therefore, the perceived ease of use is the most effective determinant that makes it possible to benefit from technology for various purposes beyond just using it. On the other hand, other psychological states or perceptions that individuals have can affect the perceived ease of use. For example, a person with high computer anxiety may not be willing to use any new technology and may not make proper use of it. Also, individuals with high emotional intelligence may believe that they can easily use the internet because they will have higher confidence in themselves and their abilities and thus, benefit from the internet for creative purposes. In this regard, the perceived ease of use may have a mediating role on the effects of computer anxiety, perceived usefulness, and emotional intelligence on internet use for creative purposes.

Dewett (2003) drew attention to the gap in the literature on the connection between technology use and creativity. After more than a decade, Oldham and Da Silva (2015) pointed out that there is still very little research directly examining the relationship between digital technology use and creative idea generation. Today, after scanning the literature, we have concluded that the gap is still there. That said, recently, Van Laar et al. (2018) developed a scale that evaluates creativity as a 21st-century digital skill and consists of items directly asking whether the individual uses the internet for creative purposes. This new tool offers an opportunity to fill the gap. Building on this recent development, the aim of this study is to link creativity to the use of digital technology, explore the key players in the process, and contribute to the literature on both creativity and technology adoption behavior. Therefore, this research aims to fill the research gaps in the literature by examining the effect of computer anxiety, emotional intelligence, and perceived usefulness on internet use for creative purposes. Moreover, the mediating role of the perceived ease of use in these relationships is also investigated. The finding enriches the literature, fills the gaps, and contributes to current knowledge.

## 2. Theoretical Background and Hypothesis Development

### 2.1. Creativity (Internet Use for Creative Purposes)

Amabile (1983) defined creativity as “the production of ideas, products or processes that are new or original and potentially useful to the organization”. However, in today’s technology-driven society, this definition of creativity is inadequate. Therefore, the use of the internet has been added to creativity, which is the process of generating ideas, solving problems, and implementing a real idea or solution in a social context, and the definition of creativity has been updated and has become a digital skill of the 21st century (Van Laar et al., 2018).

It is suggested that creative performance has three main components: domain-related skills, creativity-related processes, and task motivation. Domain-relevant skills include the full range of response possibilities from which a new response will be synthesized and the knowledge with which the new response will be evaluated. The creativity-related process determines the extent to which one’s response outperforms previous products or responses in the domain. This component starts with a specific cognitive style, characterized by the ability to understand the complexity and break the set during problem-solving, and includes heuristics for generating ideas as well as a work style that aids creative production. Task motivation refers to one’s attitude toward a task and one’s perception of why one is engaged in the task (Amabile, 2018). ICTs offer workers more autonomy and personal initiative in two different ways. First, ICTs provide a new space for personal initiative by connecting employees horizontally and increasing individuals’ participation in communication networks. Second, they facilitate the collaboration necessary to foster creativity by transcending traditional communication patterns in the hierarchy. However, fulfilling the function of making connections does not guarantee that ICTs will positively influence communication processes. This is because some individuals may be less motivated to provide information through these connections (Dewett, 2003; Oliva & Storm, 2023).

Digital tools such as e-mail and electronic conferencing allow collaboration among individuals regardless of time and place (Abendroth et al., 2024; Cabellos et al., 2024; Okoisama & Bagshaw, 2023; Zhang et al., 2024). Both knowledge-seeking and knowledge-sharing can foster creativity in the workplace, and Web 2.0 applications, such as virtual communities, offer an effective platform for this (Al-Omoush & Shuhaiber, 2024; Asghar et al., 2023; Panahi et al., 2024; Thomas, 2023). Members of virtual communities are brought together by common interests, needs, goals, or practices, and many people join these communities to seek knowledge to overcome workplace challenges (Oksanen et al., 2024). Regardless of the source of the information, it is reasonable to expect that as employees gain access to more unique and diverse information, the potential for creative combinations of this information and the likelihood of new creative ideas will increase (Oldham & Da Silva, 2015).

It is argued that there are three critical conditions necessary for employees to generate creative ideas: receiving socio-emotional or practical support, being fully engaged in one’s work and role, and having access to and exposure to new and diverse information such as ideas, methods, and perspectives. These three conditions contribute to generating creative ideas in previous studies and theories. There are strong arguments that digital technology can influence each of these three conditions. It is hypothesized that a teacher’s access to and exposure to new and diverse information will contribute to creativity by providing new ideas, different perspectives and approaches, and feed into the multicomponent process of creative idea generation. In other words, digital technology increases not only teachers’ access to information but also their ability to process and transform it into something else. It, therefore, increases the likelihood of creative ideas emerging (Oldham & Da Silva, 2015).

### 2.2. Perceived Ease of Use

Perceived ease of use can be defined as the degree of a person’s belief that using ICTs would be free of effort (Davis, 1989). This definition also involves the perception of the difficulty of learning to use a particular technology (Van Laar et al., 2019). According to Resources and Appropriation Theory, technology adoption is a sequential process called “appropriation”. Technology appropriation occurs in four access stages: attitude, material access, skills, and usage. The precondition on which this process is based is that one must first take a positive attitude towards technology to move towards the actual achievement of material access. Then, one develops the necessary skills and uses technology. The core argument of the theory is that personal and positional differences among people produce inequalities in the distribution of resources which cause inequalities in the four stages of appropriation (Van Deursen et al., 2021). The resources considered in this theory are classified under material, temporal, cultural, social, and mental resources. Materials (e.g., access to an internet connection) and temporal resources (e.g., time to use ICT) should be considered as the primary conditions for developing and using skills. Mental, social, and cultural resources can be used to explain differences in people’s abilities (Van Laar et al., 2019).

Perceived ease of use is seen as a necessary resource for the development of digital skills and as a predictor of digital skills, such as information management, information evaluation, communication expression, communication sharing, creativity, and problem-solving. Previous research has also shown that the perceived ease of use is associated with having higher levels of ICT skills and plays a strong role in the desire to develop new skills. This suggests that developing a sense of control over computers can contribute to digital skills, such as creativity (Van Laar et al., 2019). Moreover, speed and ease of use are important for users to express their creativity without being slowed down by technology. Therefore, the perceived ease of use is an important determinant of internet use for creative purposes. In addition, the individual has various feelings and thoughts that can affect the perception of ease of use, and thus, have an impact on internet use for creative purposes. In other words, perceived ease of use is seen as a potentially mediating factor in the relationship between technology use for creative purposes and its antecedents (Putro & Takahashi, 2024).

### 2.3. Computer Anxiety

Computer anxiety is described as a negative emotional or cognitive state that an individual experiences while using a computer or related equipment (Han et al., 2024). It is a type of anxiety that may comprise the fear of the unknown and frustration (Popoola & Adedokun, 2023). Individuals with computer anxiety tend to avoid the computer and are less likely to use it to its full extent (X. Li et al., 2022).

Although computers are used to improve performance in education, computer anxiety is a common phenomenon that negatively affects the performance and achievement of both teachers and students (Rahimi & Yadollahi, 2011). Computer anxiety results in computer avoidance (Cheng et al., 2023), which can be viewed from psychological, sociological, and operational perspectives. The psychological perspective focuses mainly on fear of damaging computer files and/or computer systems. The sociological perspective is interested in fear related to changes in social patterns, job requirements, and insecurity about job status due to computerization. The operational perspective includes operational problems during computer-related task fulfillment (Chua et al., 1999). 

Computer anxiety is an emotional fear and/or a reaction to potential negative consequences, such as damaging equipment or appearing incompetent. From an information processing perspective, the negative emotions associated with high anxiety interfere with the allocation of cognitive resources necessary for task performance. Therefore, individuals with high computer anxiety are expected to have lower task performance compared to individuals with less or no computer anxiety (Sam et al., 2005). 

Individuals who avoid computer use due to anxiety also lose the chance to use the opportunities offered by the computer, such as the internet. This, in turn, will limit the individual’s use of the internet for creativity. Moreover, computer anxiety can cause the individual to perceive that using the computer is difficult. Indeed, one of the main causes of computer anxiety is fear (Liu et al., 2022). A person who thinks that using the computer is difficult due to computer anxiety cannot benefit sufficiently from computers (Wu et al., 2023) and tools, such as the internet, where computers are necessary. In other words, they may not be inclined to use the internet for activities, such as searching for new ideas and information to develop their creativity. Therefore, computer anxiety may not only directly but also indirectly affect creativity. Therefore, the following hypotheses are proposed:

**H1.**  *Computer anxiety has a significant negative effect on creativity.*

**H2.**  *Perceived ease of use mediates the effect of computer anxiety on creativity.*

### 2.4. Perceived Usefulness

Perceived usefulness is one of the key factors influencing individuals’ attitudes toward the use of technology (Adu-Gyamfi et al., 2024; Davis, 1989; Kao & Huang, 2023). ICTs provide a means to manage information and knowledge (Agrawal et al., 2021; Bilan et al., 2023), which are among the most important ingredients for creativity (Dewett, 2003). Digital devices have the potential to increase the creativity of individuals’ ideas and encourage innovative thinking as they provide unique and potentially different information from a variety of sources. In addition, digital devices support the creative process by facilitating collaboration among individuals, allowing them to communicate with others who may have ideas or perspectives that are quite different from their own. Moreover, the ideas and information gathered from these individuals through digital devices can be instantly accessed, allowing them to be quickly elaborated and integrated. Furthermore, these devices provide individuals with access to a variety of resources and feedback, ensuring that information flagged as useful can be accessed and saved in a form that can be reviewed later, allowing individuals to go back and review this information during the idea development process (Salgarayeva et al., 2021). 

In contrast to technology acceptance models, the present study argues that perceived usefulness has a main effect on internet use for creative purposes and the perceived ease of use mediates this relationship. This assumption is based on the findings that the strength of the usefulness–usefulness relationship is higher than that of the ease-of-use–usefulness relationship, which treats internet use as a means to achieve creativity. Logically, if a technology is not useful for creative use, it is not suitable for a specific purpose, no matter how easy it is to use. Ease of use only facilitates the process. When a system offers basic functionality, users are often willing to tolerate some usability difficulties. While the difficulty of use can prevent the adoption of a useful system, no level of ease of use can compensate for a system that fails to perform a valuable function (Davis, 1989). The following hypotheses are, therefore, proposed:

**H3.**  *Perceived usefulness has a significant positive effect on creativity.*

**H4.**  *Perceived ease of use mediates the effect of perceived usefulness on creativity.*

### 2.5. Emotional Intelligence

Studies of the human brain show that the main criterion for measuring human intelligence is not only cognitive intelligence, but the degree of emotional intelligence is actually the most important factor in determining success in life. Emotional intelligence represents the needs, impulses, and value judgments that guide all of an individual’s behaviors and is the determinant of relationships with other individuals and success in business life (Dugué et al., 2021). The concept of emotional intelligence is derived from the idea of social intelligence, first proposed by Thorndike (1920). Thorndike defined social intelligence as “the ability to understand and manage women, men, girls, and boys in order to be rational in human relations”. Mayer et al. (1999) were among the first researchers to propose the concept of emotional intelligence to describe people’s ability to manage their own emotions. Emotional intelligence is defined as “the ability to accurately perceive, appraise, and express emotions; the ability to access or generate emotions in the thought process; the ability to understand emotions and emotional intelligence; and the ability to control emotions to enhance emotional and mental development” (Dong et al., 2022). The theorists defined emotional intelligence as a socio-emotional competency that includes the ability to monitor and discriminate between one’s own emotions and feelings, and to use this knowledge to guide one’s thinking and behavior (Van Rens et al., 2024). 

Interpersonal relationships in organizations are influenced by emotional factors rather than rational factors. Therefore, the importance of emotional intelligence should be emphasized. Emotional intelligence is important in many areas, from improving interpersonal relationships to enhancing individual performance, from facilitating team dynamics to supporting organizational culture (Dong et al., 2022). An individual’s ability to understand, connect with, and effectively manage the emotions of others can improve interpersonal communication. In fact, research has shown that emotional intelligence is associated with positive social interactions, while individuals with low emotional intelligence tend to have poorer relationships with others (Guo et al., 2024). In order for teachers to be more successful, they need to have developed emotional intelligence skills in addition to their interpersonal relationships, empathy, social skills, and the personality traits and intelligence needed to cope with challenges. This is because emotional intelligence is an integral part of teachers’ success and enhances their ability to create effective learning environments and positively influence student outcomes (Valverde-Janer et al., 2023).

Emotional intelligence can improve organizational performance by enabling the effective regulation of emotions and fostering collaboration (Dong et al., 2022). Studies on the importance of a human performance model that includes emotional intelligence criticize the theory that an individual’s knowledge, special skills, and abilities determine an organization’s performance and emphasize that emotions are more important than intellectual abilities in determining the long-term performance of employees (Sah, 2021). According to the multivariate perspective of creativity, intellectual abilities and domain knowledge, thinking styles, personality traits, and other factors interact to provide creative potential (Putro & Takahashi, 2024). Emotional intelligence can significantly enhance employees’ success in coping with stress and finding creative ways to overcome challenges (Wan et al., 2014). For example, research shows that people with high emotional intelligence tend to be more creative and positive because it enables them to overcome emotional challenges and use their emotional state to increase their creative output (Dong et al., 2022).

Based on the literature above, we propose that there is a connection between emotional intelligence and creativity. In this study, we also consider the internet as a tool for fostering creativity. Therefore, by incorporating the digital aspect, we expect the perceived ease of use to influence the relationship between emotional intelligence and creativity. Therefore, the following hypotheses are proposed:

**H5.**  *Emotional intelligence has a significant positive effect on creativity.*

**H6.**  *Perceived ease of use mediates the effect of emotional intelligence on creativity.*

**Figure 1:**
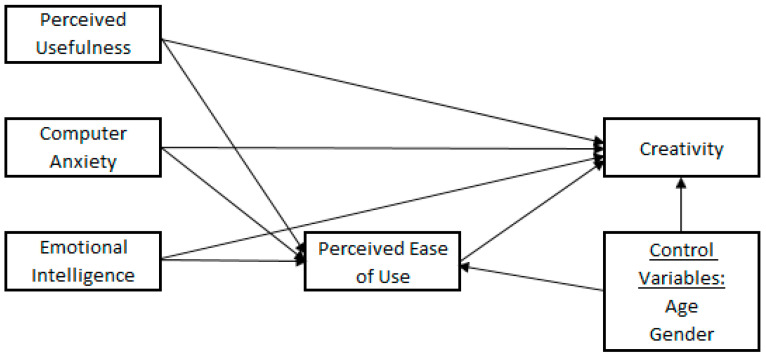
Research model.

Based on the discussion above, the proposed research model is given in Figure 1.

## 3. Method 

### 3.1. Reseacrh Design

This research employed a cross-sectional design to examine the effect of computer anxiety, emotional intelligence, and perceived usefulness on internet use for creative purposes with the mediating role of the perceived ease of use. A cross-sectional design is a research design in which data about dependent and independent variables are collected at the same time (Jiang & Messersmith, 2018). In this regard, a questionnaire was prepared that included various demographic information and scales measuring variables. These scales were adopted from previous research, namely, they are prevalidated scales. All scales were included in the same survey and participants responded to the survey within a specific time.

We carried out an online survey to examine the hypothesized relationships. The only criterion for participation in our study was to be a full-time employee at an educational institution. Starting with the principal investigator’s initial contact dataset, we sent the URL link to the online survey to full-time staff at various educational institutions and made it clear that no information collected through the online survey would be disclosed to anyone. We adopted a snowball strategy and asked participants to distribute the URL link among their contacts who were also full-time employees at an educational institution.

### 3.2. Context and Participants

The internet has become a frequently used tool in the field of education, especially after the pandemic, and enables both access to information and various forms of learning. Moreover, it has become a necessity for teachers to use the internet for creative purposes and to be competent in this regard (Y. Li et al., 2023). In this context, this research that investigates the antecedents of internet use for creative purposes was conducted with teachers in the Marmara Region in Turkey.

The snowball sampling technique, which is a very effective method for including hard-to-reach subjects in the sample, uses the social network connections of the participants. Since these social network connections will consist of people like the participants themselves, it may cause sampling bias and a margin of error. To avoid this, the sample size was increased in the study and attention was paid to the demographic characteristics of the participants. A total number of 264 individuals participated in the survey. When the data were checked, it was observed that there were no missing data. Here, 56.4% of them were female and the average age was 37.15 years (SD = 7.36). The graduation levels of participants were 26.2% with a bachelor’s degree, 42.4% with a master’s degree, and 31.4% with a doctoral degree.

### 3.3. Measurement Scales

To measure variables, we adopted scales from previous studies since they have been used in many studies and their reliability has been proven many times. Emotional intelligence, computer anxiety, perceived usefulness, and perceived ease of use (PEOU) were measured using a 5-point Likert scale from 1 (Strongly disagree) to 5 (Strongly agree). The internet use for creative purposes was measured using a 5-point Likert scale (1 = never, 2 = rarely, 3 = sometimes, 4 = often, 5 = always). 

The emotional intelligence (EI) scale was adapted from Wong and Law (2017). The 16-item emotional intelligence scale consists of four dimensions containing 4 items each: self-emotion appraisal (SEA), others’ emotion appraisal (OEA), use of emotion (UOE), and regulation of emotion (ROE). In this research, the internal consistency (Cronbach alpha) value ranged from 0.823 to 0.883 (see Table 1).

For computer anxiety (CA), 4 items were used from the scale of Thatcher and Perrewe (2002), which originated from the Computer Anxiety Scale (CAS) developed by Heinssen et al. (1987). In this research, the internal consistency (Cronbach alpha) value was 0.889.

We measured perceived usefulness (PU) of the internet with the 6-item perceived usefulness scale of Davis (1989). In this research, the internal consistency (Cronbach alpha) value was 0.940.

We measured the perceived ease of use (PEOU) of the internet with the 6-item perceived ease of use scale of Davis (1989). In this research, the internal consistency (Cronbach alpha) value was 0.918.

We measured the internet use for creative purposes (UC) with the 6-item Creativity Scale, which was developed by Van Laar et al. (2018) to evaluate creativity as a 21st-century digital skill, consisting of items directly asking whether the individual uses the internet for creative purposes. A sample item was “I give a creative turn to existing processes using the internet”. In this research, the internal consistency (Cronbach alpha) value was 0.892.

This study used age and gender (1 = female, and 2 = male) as control variables. Research in literature with technology-related variables in general indicates that gender and age could influence our research model; therefore, we decided to include them.

### 3.4. Data Collection

We carried out an online survey to examine the hypothesized relationships. The only criterion for participation in our study was to be a full-time employee at an educational institution. Starting with the principal investigator’s initial contact dataset, we sent the URL link to the online survey to full-time staff at various educational institutions and made it clear that no information collected through the online survey would be disclosed to anyone. We adopted a snowball strategy and asked participants to distribute the URL link among their contacts who were also full-time employees at an educational institution. The snowball sampling technique, which is a very effective method for including hard-to-reach subjects in the sample, uses the social network connections of the participants. Since these social network connections will consist of people like the participants themselves, it may cause sampling bias and a margin of error. To avoid this, the sample size was increased in the study and attention was paid to the demographic characteristics of the participants. A total number of 264 individuals participated in the survey. When the data were checked, it was observed that there were no missing data. Here, 56.4% of them were female and the age average was 37.15 years (SD = 7.36). The graduation levels of participants were 26.2% with a bachelor’s degree, 42.4% with a master’s degree, and 31.4% with a doctoral degree.

## 4. Data Analysis and Results

### 4.1. Measurement Validity and Reliability

First, we performed a Confirmatory Factor Analysis (CFA) containing all variables in the research model: Computer anxiety, perceived usefulness, the perceived ease of use, creativity, and emotional intelligence dimensions, SEA, OEA, ROE, UOE. Analysis indicated a good model fit: χ^2^/df = 1.656 < 3, RMSEA = 0.050 < 0.08, CFI = 0.945 > 0.9, IFI = 0.945 > 0.9, TLI = 0.938 > 0.9, SRMR = 0.046 < 0.05. Factor loadings are shown in Table 1. 

After CFA, reliability, and validity were tested. The Composite Reliability (CR) and Average Variance Extracted (AVE) values were used to assess the convergent and discriminant validity. CR values of all variables were all above 0.7 (in the range of 0.834–0.942) and AVE values of all constructs were greater than the cut-off value of 0.50 (Table 1), which indicates good convergent validity (Fornell & Larcker, 1981). The AVE square root for each construct was consistently greater than the correlation coefficient between the constructs (Table 2), which indicates a high discriminant validity (Fornell & Larcker, 1981). Moreover, to test reliability, Cronbach’s α coefficients were computed. It is found that Cronbach’s α coefficients of all variables in this study were greater than 0.8 (Hair et al., 2013), indicating reliability. These results mean that the measurement model of the present study is valid and reliable. 

Then, we performed a correlation analysis with all variables and its findings are shown in Table 2.

### 4.2. Hypothesis Testing

**Figure 2:**
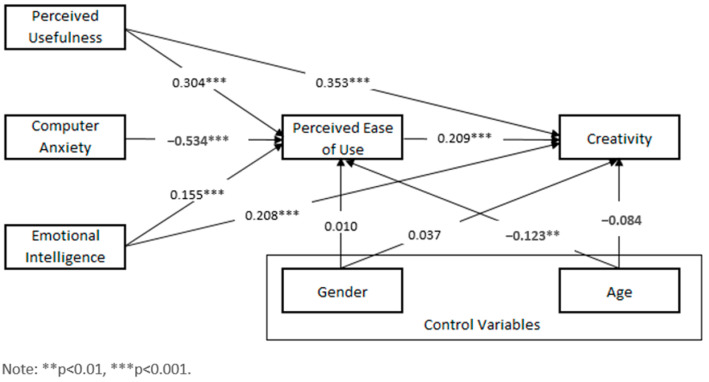
Empirical study results.

We used structural equation modeling (SEM) to evaluate our research model. The standardized path coefficients are shown in Figure 2, and the analysis indicated a good model fit: χ^2^/df = 1.491 < 3 (χ^2^ = 1.491, df = 1, *p* = 0.222), RMSEA = 0.043 < 0.08, CFI = 0.998 > 0.9, IFI = 0.998 > 0.9, TLI = 0.965 > 0.9, SRMR = 0.009 < 0.05. According to results, all direct associations were found to be statistically significant. 

We also performed bootstrapping (2000 samples, %95 confidence level) to test mediation hypotheses. The path analysis results for all direct and indirect effects related to the paths included in our model are shown in Table 3. Moreover, the estimates presented in Table 3 are unstandardized. 

As seen in Table 3, the findings confirm the intermediary role of PEOU in the correlation among independent variables (CA, PU, and EI) and UC. In detail, according to the results, PEOU has a partial mediating role in the relationship between EI and UC and in the relationship between PU and UC. On the other hand, PEOU has a full mediating effect on the relationship between CA and CU.

## 5. Discussion and Implications 

Considering the lack of research examining the concept of creativity and its antecedents in terms of the use of digital technologies, this study attempts to fill this gap and examine the technology acceptance model and creativity together. The primary aim of this study was to examine the factors that predict the use of internet for creativity. While doing this, we used the perceived ease of use as a general intermediary factor, which has been shown in many studies to play a mediating role between external factors and actual technology use in the technology acceptance model. The results of analyses supported all the hypotheses of the study, and our overall research model has proven to have efficient model fit values.

First, it is found that computer anxiety is negatively related to internet use for creative purposes. Previous studies have shown that computer anxiety negatively affects attitudes toward computers and activities that require computers. For example, Rezaeı et al. (2008) revealed in their study that the internet and computer technologies are a tool that improves creativity, while the perception of technology as complex and computer anxiety negatively affect the intention to use it. Cazan et al. (2016) found that computer anxiety affects negative attitudes towards internet use. That is, computer anxiety causes the individual to develop a negative attitude towards the computer and avoid using it. In addition, people with computer anxiety also avoid using the computer for whatever purpose. In this context, X. Li et al. (2022) stated that people with computer anxiety are less likely to use the online learning system. Rahimi and Yadollahi (2011) also found that teachers’ computer anxiety has a negative relationship with the integration of information and communication technologies into English lessons. Therefore, although there is no study investigating the association between computer anxiety and internet use for creative purposes specifically, our finding is in line with previous studies. This result may be due to the fact that people with computer anxiety cannot learn to use the computer fully and benefit from it (Wu et al., 2023). In other words, in addition to avoiding tasks to be performed using the computer, they cannot perform them with high efficiency even if they have to. Therefore, people with computer anxiety avoid using the internet, do not go beyond necessity even if they have to use it, and thus, they are unlikely to use the internet for creative purposes. That is, this finding supports previous research and extends existing knowledge by providing empirical evidence.

Second, findings demonstrate that perceived usefulness affects internet use for creative purposes positively. Similarly, Lymperopoulos and Chaniotakis (2005) found that the perceived usefulness of the internet directly affects employees’ attitudes, which in turn, affects their intention to use the internet as a tool. Cho et al. (2020), in their study on the perceived usefulness and perceived ease of use of information and communication technologies, revealed that it affects the participation of older adults in activities. Gunasekara et al. (2022) found that perceived usefulness positively affects attitudes towards internet use for health-related purposes. Thus, our result is in line with previous studies. On the other hand, this finding indicates that if people perceive the usefulness of something, they will tend to use it, and they can obtain beneficial results by using it for many purposes. Conversely, if they think it is not useful, they may not want to spend time with it and may not make an effort to benefit from it (Kao & Huang, 2023). This is because people believe that if the perceived usefulness is high, they will achieve higher performance when they use it for a specific purpose (Davis, 1989). In other words, only people with high perceived usefulness prefer to use the internet for creative purposes, since when this perception is low, namely, when it is thought that no useful results can be obtained from it, using the internet for creative purposes may be seen as unnecessary and a waste of time. Therefore, this finding reveals the critical role of perceived usefulness for internet use for creative purposes.

Third, it is demonstrated that emotional intelligence has a positive effect on internet use for creative purposes. Since past studies have shown that emotional intelligence positively affects creativity and creative performance (Shafait & Huang, 2024; Silva & Coelho, 2019; Stawicki et al., 2023), this finding is in line with previous research. Emotional intelligence has significant positive effects on all kinds of work-related activities. Even when cognitive intelligence is low, if emotional intelligence is high, motivation and commitment to work can be high, the quality of decision-making can increase, and a way can be found to be successful by being aware of deficiencies (Dong et al., 2022). Emotional intelligence allows individuals to have higher adaptability and higher motivation. (Guo et al., 2024). Therefore, individuals with high emotional intelligence are more productive at work than others and they can manage work-related stress in the best way (Dugué et al., 2021). In other words, people with high emotional intelligence do not hesitate to use the internet and even tend to use it to do their job better. In addition, creativity is an extra-role behavior. In this case, people need to be in a positive emotional state towards their work to engage in extra-role behavior, such as creativity for their work, and emotional intelligence allows people to cope with negativities and be in a positive emotional state. For this reason, people with high emotional intelligence tend to be creative in their work (Wan et al., 2014). That is, since people with high emotional intelligence have a positive work-related attitude, they want to be beneficial to the work results and also have not any negative attitude toward the internet. As a result, they are more inclined to use the internet for creative purposes. This finding offers a new perspective by revealing the effect of emotional intelligence on internet use for creative purposes. 

Finally, we found that perceived ease of use mediates the effects of computer anxiety, perceived usefulness, and emotional intelligence on internet use for creative purposes. No matter how easy a technology is to use, it will not be preferred if it does not serve its purpose (Radner & Rothschild, 1975; Davis, 1989; Teo et al., 1999). This will reveal the fact that the perception of usefulness should be prioritized, and ease of use will play a secondary role as a factor contributing to the process. Luo et al. (2024) concluded that perceived ease of use and perceived usefulness are the main factors necessary for the adoption of health information systems. In other words, unlike other studies on this subject, in this study, perceived usefulness was considered as the first condition of using the internet for creative purposes, and the mediating role of the perceived ease of use in this relationship was examined. This issue was addressed by Urban et al. (2024) in the form of students’ developing creative problem solutions through chatbots in terms of perceived usefulness and ease of use. Logically, if technology is to be used for a specific purpose, firstly, a technology that will serve this purpose should be selected. Then, the technology that will require the least effort among the available options will be selected because difficulty can increase computer anxiety in individuals. Naudé et al. (2024) revealed in their study that difficulty in use increases computer anxiety. On the other hand, the approaches of people with or without high emotional intelligence to perceived ease of use can affect creativity. Abu-Shanab and Shanab (2022) stated that emotional intelligence is a driving force in technology adoption. Finally, all variables were considered as a whole, and it was found that perceived ease of use mediated the effects of computer anxiety, perceived usefulness, and emotional intelligence on internet use for creative purposes.

Our results confirmed that teachers’ perceived usefulness of the internet and emotional intelligence both have a positive impact, while computer anxiety has a negative impact on internet use for creative purposes. In addition, this study showed that the perceived ease of use of the internet plays a mediating role in the relationship between all three variables and internet use for creative purposes. It is noteworthy that it plays a complete intermediary role in the correlation between computer anxiety and creativity. Our results have contributed to the creativity literature in terms of adding a digital aspect to creativity and examining its antecedents in this direction. In addition, it has replaced the use of technology at the endpoint of the technology acceptance model with creativity, opening the door to new research possibilities where this model can be used. At this point, for future research, it is aimed to draw attention to the digital competencies for the 21st century identified by Van Laar et al. (2018), who updated the variables frequently used in the literature by bringing a digital perspective.

Practically, research findings also offer important tips that are empirically proven on ways to increase internet use for creative purposes. The results show that computer anxiety is an important obstacle to achieving this. Therefore, various trainings can be organized to increase the knowledge of employees about using computers and thus, this anxiety can be eliminated. Perceived usefulness and emotional intelligence stand out as important factors that enable internet use for creative purposes. Managers should provide information about the usefulness of the internet, support creative work performed using the internet and thus, support the increase in perception of usefulness. In addition, emotional intelligence should be an important criterion in recruitment and policies should be developed to increase the emotional intelligence of current employees. Finally, it is revealed that perceived ease of use has a mediating role in the effect of all these antecedents on internet use for creative purposes. In this regard, managers should pay attention to ease of use when planning and maintaining other applications for internet use for creative purposes.

## 6. Conclusions, Limitations, and Recommendations

The internet is used by almost everyone all over the world. For the business world, its use has become a necessity. However, using it for various purposes beyond standard use is important in terms of gaining maximum efficiency from it and in terms of the employee’s job performance. However, access to information has become very easy due to the advantages provided by the internet. For this reason, one of the most important factors that create differences in employees is considered to be the use of the internet for creative purposes. In this context, this study aims to reveal the factors that affect internet use for creative purposes. The findings show that computer anxiety negatively and perceived usefulness and emotional intelligence positively affect internet use for creative purposes. Moreover, it was found that perceived ease of use has a mediating effect on these effects. Therefore, this study contributes to the existing literature and offers important managerial implications.

However, like every empirical study, this study also has various limitations. The main limitation of this study was that the examinations were conducted by selecting a specific business sector (education). Although this study has established a relationship between the use of the internet for creativity for education and its predecessors, it may be expected to yield different results for individuals working in different sectors. In addition, the concept of the internet is used as an umbrella term today and includes many different technological tools. Studies for specific digital tools, such as YouTube, forums, chat environments, Research Gate, etc., may contribute to explaining these relationships better. Moreover, other variables, such as trust, support, and perceived risk, may be added to the research model and tested in future research.

## Figures and Tables

**Table 1:** The measurement model statistics.

Variable	Items	Loading	Cronbach’s α	CR	AVE
Self-emotion appraisal (SEA)	SEA1	0.784	0.856	0.858	0.668
	SEA2	0.833			
	SEA3	0.834			
Others’ emotion appraisal (OEA)	OEA1	0.804	0.878	0.880	0.709
	OEA2	0.874			
	OEA4	0.847			
Use of emotion (UOE)	UOE2	0.707	0.823	0.834	0.628
	UOE3	0.881			
	UOE4	0.779			
Regulation of emotion (ROE)	ROE1	0.810	0.883	0.884	0.657
	ROE2	0.851			
	ROE3	0.735			
	ROE4	0.842			
Computer anxiety (CA)	CA1	0.831	0.889	0.894	0.679
	CA2	0.814			
	CA3	0.840			
	CA4	0.810			
Perceived usefulness (PU)	PU1	0.814	0.940	0.942	0.730
	PU2	0.827			
	PU3	0.917			
	PU4	0.877			
	PU5	0.904			
	PU6	0.778			
Perceived ease of use (PEOU)	PEOU1	0.866	0.918	0.924	0.670
	PEOU2	0.841			
	PEOU3	0.803			
	PEOU4	0.709			
	PEOU5	0.796			
	PEOU6	0.883			
Creativity (UC)	UC1	0.787	0.892	0.896	0.591
	UC2	0.828			
	UC3	0.843			
	UC4	0.817			
	UC5	0.664			
	UC6	0.649			

**Table 2:** Correlation coefficients and descriptive statistics.

Variable	Mean	SD	1	2	3	4	5	6	7	8
1. Self-emotion appraisal	4.36	0.59	(0.817)							
2. Others’ emotion appraisal	3.97	0.68	0.396 **	(0.842)						
3. Use of emotion	3.81	0.80	0.363 **	0.204 **	(0.792)					
4. Regulation of emotion	3.56	0.83	0.310 **	0.282 **	0.496 **	(0.811)				
5. Computer anxiety	1.63	0.86	−0.063	−0.008	−0.094	−0.055	(0.824)			
6. Perceived ease of use	4.51	0.56	0.166 **	0.162 **	0.196 **	0.167 **	−0.593 **	(0.818)		
7. Perceived usefulness	4.65	0.50	0.087	0.143 *	0.108	0.144 *	−0.111	0.394 **	(0.854)	
8. Creativity	4.13	0.70	0.203 **	0.187 **	0.274 **	0.227 **	−0.242 **	0.415 **	0.473 **	(0.769)
Age	37.15	70.36	0.055	−0.062	0.164 **	0.049	0.090	−0.170 **	−0.037	−0.113
Gender	-	-	−0.048	−0.046	0.065	0.045	−0.196 **	0.095	−0.034	0.040

* *p* < 0.05; ** *p* < 0.01; diagonals show the square roof of AVEs.

**Table 3:** Path analyses of direct and indirect effects.

Path		Estimate	Lower	Upper	*p*
Direct Effects					
	CA-->PEOU	−0.345	−0.429	−0.261	0.001
	PU-->PEOU	0.338	0.225	0.464	0.001
	Gender-->PEOU	0.011	−0.084	0.108	0.794
	Age-->PEOU	−0.009	−0.017	−0.003	0.003
	EI-->PEOU	0.163	0.076	0.264	0.001
	PU-->UC	0.496	0.330	0.670	0.001
	Gender-->UC	0.052	−0.086	0.196	0.443
	Age-->UC	−0.008	−0.017	0.001	0.094
	PEOU-->UC	0.263	0.123	0.419	0.001
	EI-->UC	0.276	0.147	0.413	0.001
Indirect Effects					
	PU-->PEOU-->UC	0.089	0.039	0.166	0.001
	CA-->PEOU-->UC	−0.091	−0.151	−0.042	0.001
	EI-->PEOU-->UC	0.043	0.016	0.094	0.000

Notes. Estimates are not standardized. Bootstrapping with 2000 samples and 95% confidence level.

## Data Availability

The data presented in this study are available on request from the corresponding author.
